# Mitigation effects of plant carbon black on intestinal morphology, inflammation, antioxidant status, and microbiota in piglets challenged with deoxynivalenol

**DOI:** 10.3389/fimmu.2024.1454530

**Published:** 2024-09-09

**Authors:** Jie Wu, Hanyang Wang, Jianling Liao, Linfu Ke, Deqiu Lu, Bo Deng, Ziwei Xu

**Affiliations:** ^1^ Institute of Animal Husbandry and Veterinary Science, Zhejiang Academy of Agricultural Sciences, Hangzhou, China; ^2^ College of Animal Science and Technology, Northeast Agricultural University, Harbin, China; ^3^ Department of Techniques Developing, Fujian Baicaoshuang Biotechnology Co., Ltd., Nanping, China; ^4^ Department of Production Research and Development, Harbin PuFan Feed Co., Ltd., Harbin, China

**Keywords:** deoxynivalenol, piglets, plant carbon black, intestinal morphology, inflammation, antioxidant status, gut microbiota

## Abstract

**Introduction:**

Plant carbon black (PCB) is a new feed additive for zearalenone adsorption in China. However, information regarding whether PCB can effectively absorb deoxynivalenol (DON) is limited.

**Methods:**

To explore this research gap, the present study examined the adsorption effectiveness of DON by PCB using a phosphate buffer, artificial gastric juice, and artificial intestinal juice. In a 21-day *in vivo* trial, 48 male piglets were randomly assigned to four treatment groups: (1) uncontaminated basal diet (CTR), (2) basal diet supplemented with 1 mg/kg PCB(PCB), (3) 2.3 mg/kg DON-contaminated diet (DON), and (4) 2.3 mg/kg DON-contaminated diet supplemented with 0.1% PCB (DON+PCB).

**Results:**

When DON concentration was 1 µg/mL, the adsorption rate of PCB on DON in phosphate buffer systems (pH 2.0 and 6.0) and the artificial gastric and intestinal juices were 100%, 100%, 71.46%, and 77.20%, respectively. In the *in vivo* trial, the DON group significantly increased the DON+deepoxy-deoxynivalenol (DOM-1) content in serum as well as the inflammation cytokine proteins (interleukin-6, interleukin-8, and tumor necrosis factor-α) and mRNA expression of interleukin-6 and longchain acyl-CoA synthetase 4 in the jejunum and ileum. It decreased the villus height, goblet cells, mucosal thickness, and mRNA expression of Claudin-1 compared to the CTR group. In addition, DON decreased the Shannon and Simpson indices; reduced the relative abundances of *Firmicutes*, *Lactobacillus*, *Candidatus_Saccharimonas*, and *Ruminococcus*; and increased the relative abundances of *Terrisporobacter* and *Clostridium_sensu_stricto_1* in the cecal content.

**Discussion:**

In conclusion, these results suggest that PCB showed high adsorption efficacy on DON *in vitro*, and exhibit the protective effects against various intestinal toxicity manifestations in DON-challenged piglets.

## 1 Introduction

Deoxynivalenol (DON), commonly referred to as vomitoxin, is a mycotoxin produced by multiple *Fusariums*, including *F. graminearum, F. cerealis*, and *F. culmorum*, which extensively contaminate crops throughout various stages such as growth, harvest, storage, and processing (1). A survey incorporating 9,239 feed samples noted that DON emerged as a predominant feed-associated mycotoxin in China from 2017 to 2021, with a positive rate of 87.07% and an average of 838.89 μg/kg (2). Pigs are considered the most sensitive farm animals to DON (3). Increasing evidence has indicated that exposing pigs to feed contaminated with DON results in a range of toxic symptoms such as vomiting, diarrhea, reduced food intake, body weight loss, and immunosuppression (4–6).

DON is absorbed and metabolized in the upper part of the small intestine, making the intestinal epithelium the primary target of DON (7). DON exposure can trigger inflammatory responses, alter intestinal morphology, and disrupt intestinal tight junctions, destroying the intestine’s physical barrier and immune functions (8–10). Moreover, DON induces oxidative stress by overproducing reactive oxygen species that can counteract antioxidant defense systems (11, 12). Thus, reducing intestinal damage is crucial in mitigating the subsequent toxicity of DON to other tissues.

To mitigate the risks posed by mycotoxin-contaminated feeds, mycotoxin adsorbents have been extensively studied as feed additives that bind mycotoxins within the gastrointestinal environment. The complex adsorbing mycotoxins pass through the intestines and are then excreted in the feces, reducing mycotoxins’ bioavailability and subsequent toxicity. Numerous studies have shown the effectiveness of mycotoxin binders, particularly aflatoxins, in mitigating intestinal mycotoxin exposure and reducing subsequent toxicity (13, 14). However, binding and mitigating the effects of DON pose challenges for several mycotoxin adsorbent products because of DON’s high polarity, lack of coplanar structure, and low molecular weight (15). Even in *in vitro* buffer systems, most commercial products fail to bind DON with an absorption rate of less than 20% (16).

Activated carbon is an insoluble powder created through the carbonization of nearly any organic material, followed by an activation procedure to enhance its adsorption capacity. Encouraging findings have demonstrated that activated carbon effectively adsorbs DON *in vitro* (16, 17). However, not all activated carbons can adsorb mycotoxins adequately, and this adsorption efficacy depends on the source material, pore size distribution, and surface area (18). Moreover, the *in vivo* adsorption efficacy is reduced or ineffective due to animal age and mycotoxin concentration (19, 20). Plant carbon black (PCB), or baicaoshuang, is a traditional Chinese herbal medicine composed mainly of porous carbon derived from herbal plants. PCB belongs to the activated carbon class of adsorbents but has a larger surface area and a more suitable pore size (21). PCB is commonly used as a colorant in the food industry and was approved by the Ministry of Agriculture of China in 2020 as a feed additive for adsorbing zearalenone (ZEN). However, the DON adsorption efficacy of PCB requires further investigation. In this study, we investigated the adsorption of DON using an *in vitro* buffer system to explore this research gap. We then assessed the protective effects of PCB on the intestinal morphology, inflammation, antioxidant capacity, and gut microbiota of DON-challenged piglets.

## 2 Materials and methods

### 2.1 DON and PCB information

DON (purity ≥ 98%) was acquired from TripleBond Scientific Inc. (Guelph, CA, Canada). PCB was acquired from Fujian Baicaoshuang Biotechnology Co., Ltd. (Nan Ping, Fujian, China).

### 2.2 DON adsorption experiment *in vitro*

**Figure 1:**
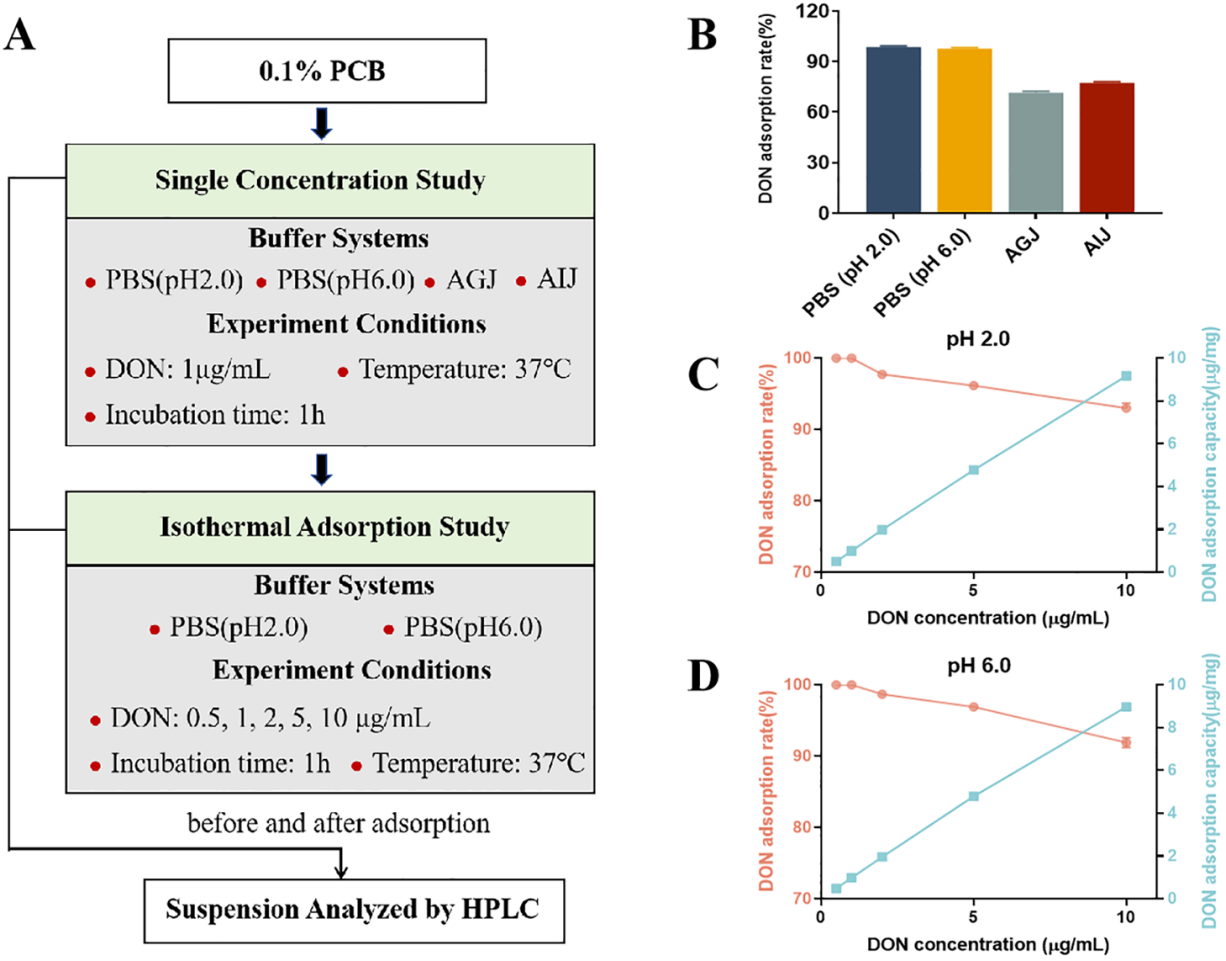
The adsorption of deoxynivalenol (DON) by plant carbon black (PCB) under different parameters. **(A)** The explanatory scheme for the *in vitro* experimental design. **(B)** Adsorption rate of DON (1 μg/mL) by 1 mg/mL PCB under different buffer systems. **(C)** Adsorption rate and capacity of different initial concentrations of DON by 1 mg/mL PCB in PBS buffer (pH 2.0). **(D)** Adsorption rate and capacity of different initial concentration of DON by 1 mg/mL PCB in PBS buffer (pH 6.0). *N* = 3. PBS, phosphate buffered saline; AGJ, artificial gastric juice; AIJ, artificial intestinal juice. HPLC, high-performance liquid chromatography.

The *in vitro* experiment consisted of two parts (**Figure 1A**): a single-concentration study and an adsorption isotherm study. For the single-concentration study, four buffer systems were employed to simulate different gastrointestinal conditions: phosphate buffer at pH 2.0 and 6.0, artificial gastric juice (AGJ, pH 2.0, consisting of 0.32% pepsin and 0.2% NaCl), and artificial intestinal juice (AIJ, pH 6.5, consisting of 1% trypsin and 0.68% KH_2_PO_4_). The preparation method of AGJ and AIJ is shown in **Supplementary Method S1**. In each system, 10 mg of PCB was added into 10 mL of buffer containing 1 μg/mL DON. The mixtures were shaken by an oscillation shaker at 37°C for 1 h, followed by centrifugation at 1,500*g* for 5 min to separate PCB from the suspension. The adsorption isotherm study followed an identical protocol, with the key variation being the initial DON concentrations (0.5, 1, 2, 5, and 10 μg/mL) in phosphate buffers at pH of 2.0 and 6.0. All adsorption experiments were performed in triplicate. Suspensions before and after the adsorption process were collected, and the DON content was determined as previously described (16). The equations below were employed to determine the adsorption capacity of PCB for DON (μg/mg) and the DON adsorption rate (%).


$$\text{DON adsorption capacity }\left(\text{μg/mg}\right)\text{ = }\frac{\text{(C}0-\text{C) V}}{\text{m}}$$

$$\text{DON adsorption rate }\left(\%\right)\;=\;\frac{C0-C}{\text{C}0}\;\times \;100\%$$

where C0 and C represent the DON concentrations in the suspension (μg/mL) before and after the adsorption process, respectively.

### 2.3 *In vivo* experimental design and animal management

In this 21-day trial, 48 male castrated crossbred (Duroc × Large white × Landrace) piglets with an initial body weight of 7.76 ± 0.24 kg and an age of 24 days were utilized. All piglets were divided into four groups, each with three replicates and four piglets per replicate. The four treatments included an uncontaminated basal diet (CTR), a basal diet + 0.1% PCB (PCB), a 2.3 mg/kg DON-contaminated basal diet (DON), and a 2.3 mg/kg DON-contaminated basal diet (DON) + 0.1% PCB (DON+PCB). Basal diets were offered in meal form, and the ingredients and nutrient composition are shown in **Supplementary Table S1**. To ensure the uniform distribution of DON in the contaminated diets, we used a stepwise dilution method. First, DON was dissolved in water and evenly sprayed on corn meal. Then, the DON-containing corn meal was gradually diluted into a 1% premix, followed by a 4% premix. Finally, the 4% premix was incorporated into the complete feed. The levels of mycotoxins in the feed were determined using liquid chromatography, as described previously (22) (**Supplementary Table S2**).

All piglets had unlimited food and water availability during the entire trial.

### 2.4 Sample collection

At 8:00 a.m. on the 22nd day, two healthy piglets were selected from each pen for blood sampling. Serum samples were obtained by centrifugation at 2,000*g* for 10 min. After blood sampling, the piglets were euthanized via intravenous injection with Zoletil and exsanguination.

Middle jejunum and terminal ileum samples were collected and washed with ice-cold physiological saline. To perform a histological analysis, the jejunal and ileal segments were post-fixed in 4% paraformaldehyde, and the jejunal segment was fixed in 2.5% glutaraldehyde. For enzyme activity analysis and RNA extraction, mucosal samples were collected using slides and then rapidly frozen in liquid nitrogen. Cecal contents were collected and rapidly frozen in liquid nitrogen for 16S rRNA and short-chain fatty acid (SCFA) measurements.

### 2.5 Analysis of DON and deepoxy-deoxynivalenol in serum

To prepare the serum samples, 5 mL of 0.1% formic acid-acetonitrile solution was added to 2.0 mL of serum sample and vortexed for 2 min. After refrigeration for 15 min, 0.2 g of NaCl and 0.8 g of anhydrous MgSO_4_ were added and vortexed for 1 min. After centrifugation at 3000*g* for 5 min, the supernatant was taken, and 600 mg anhydrous MgSO_4_ and 100 mg each of C18, PSA, and A-AL were added. After vortexing for 1 min, the supernatant was filtered through a 0.22μm nylon microporous filter membrane after centrifugation and vacuum concentrated to dryness. The residue was dissolved in 0.5 mL of acetonitrile for measurement. Serum DON and DOM-1 levels were analyzed using HPLC-MS/MS (SCIEX 5500+, SCIEX, MA, USA), as previously described (23).

### 2.6 Antioxidant parameters and inflammation cytokines in the jejunum and ileum mucosa

The levels of total antioxidant capacity (T-AOC), superoxide dismutase (SOD), catalase (CAT), malondialdehyde (MDA), glutathione peroxidase (GSH-Px), interleukin-1β (IL-1β), IL-6, IL-8, IL-10, tumor necrosis factor-α (TNF-α), diamine oxidase (DAO), myeloperoxidase (MPO), and inducible nitric oxide synthase (iNOS) in the jejunum and ileum were detected according to the commercial kits’ instructions (Nanjing Jiancheng Bioengineering Institute, Nanjing, China).

### 2.7 Intestinal morphology and ultrastructure analysis

After fixation in 4% paraformaldehyde for 48 h, the intestines were embedded in paraffin. Sections (0.5 mm) were stained with hematoxylin and eosin (HE) to analyze villus height, crypt depth, and mucosal thickness, as well as with Alcian blue-PAS (AB-PAS) to analyze goblet cells, according to a previous study (24).

The ultrastructural morphology of jejunum microvilli was observed via scanning electron microscopy (SEM, JSM-6490LV, JEOL, Tokyo, Japan) and transmission electron microscopy (TEM, H-7650, Hitachi, Tokyo, Japan) as previously described (25).

### 2.8 RNA extraction and qPCR

Mucosal RNA was isolated using RNAiso Plus kits (Takara, Tokyo, Japan). Total RNA (1 μg) was quantified and reverse-transcribed to cDNA using a TAKARA PrimeScript Kit (Takara, Tokyo, Japan). Quantitative PCR was performed using an SYBR Premix Ex Taq (Takara, Tokyo, Japan) in an ABI Plus One RT-PCR System (Life Technologies, Carlsbad, CA, USA). The PCR cycling parameters included an initial denaturation step at 95°C for 34 s, followed by 40 cycles at 95°C for 5 s and 62°C for 34 s. The sequences of primers are presented in **Supplementary Table S3**.

### 2.9 Measurement of SCFA

Cecal SCFA concentrations, including acetic, propionic, butyric, and isovaleric acids, were tested using an Agilent model 7890 GC (Agilent, CA, USA) with a flame ionization detector and capillary Nukol column (Supelco, Bellefonte, PA, USA) according to our previous study (26).

### 2.10 16S rDNA sequencing and cecal microbiota analysis

Total genome DNA from the cecal chyme was extracted using a MicroElute Genomic DNA kit (D3096-01; Omega Inc., USA). The amplification of the V3–V4 region of 16S rRNA was performed using primers 319F-806R (forward 319F: 5′-ACTCCTACGGGAGGCAGCAG-3′ and reverse 806R: 5′-GGACTACHVGG GTWTCTAAT-3′). Raw data were processed using Greengenes, and the Ribosomal Database Project database (version 13.8) was used for sequence analysis, comparison, and annotation. Sequencing was carried out using an Illumina MiSeq 2 × 300 bp paired-end. In R version 3.2.3 (RStudio Team, Boston, MA, USA), a similarity limit of 97% was established for operational taxa (OTUs). In addition, α-diversity and β-diversity analyses of the cecal microbiota were performed by QIIME 1.7.0-dev-based (Rob Knight; Fort Collins, CO, USA).

### 2.11 Statistical analysis

All data except the microbiota were analyzed as a 2×2 factorial arrangement using SPSS (version 22.0; SPSS Inc., Chicago, IL, USA). The model included the effects of DON and PCB and their interactions. Differences among the groups were further compared by ANOVA using an LSD multiple comparison test. We used R Version 3.2.3, relevant R software packages, and Qiime 1.7.0 for all the statistical analyses and data visualizations to facilitate a microbiota analysis. Correlations between microbiota and protein expression in the jejunum and SCFA were analyzed using Pearson’s correlation using R statistical software. Data were presented as means ± SEM. Pigs selected from each pen were considered experimental units for the other indices. Statistically significant differences were considered at *p* < 0.05.

## 3 Results

### 3.1 DON adsorption of PCB *in vitro*

The *in vitro* study showed that PCB completely adsorbed DON in phosphate buffer systems with a pH of 2.0 or 6.0 and a DON concentration of 1 μg/mL (**Figure 1B**). In artificial gastric and intestinal juices, the adsorption rates of PCB for DON decreased to 71.46% and 77.20%, respectively (**Figure 1B**). The adsorption isotherm studies revealed similar isothermal adsorption behavior at both pH 2.0 (**Figure 1C**) and 6.0 (**Figure 1D**). As the DON concentration increased from 1 to 10 μg/mL, a slight decrease in adsorption rate was observed: from 100% to 93.02% at pH 2.0, and from 100% to 91.92% at pH 6.0. Concurrently, the DON adsorption capacity increased: from 0.508 to 9.174 μg/mg at pH 2.0, and from 0.489 to 8.972 μg/mg at pH 6.0.

### 3.2 DON residue in serum

**Figure 2:**
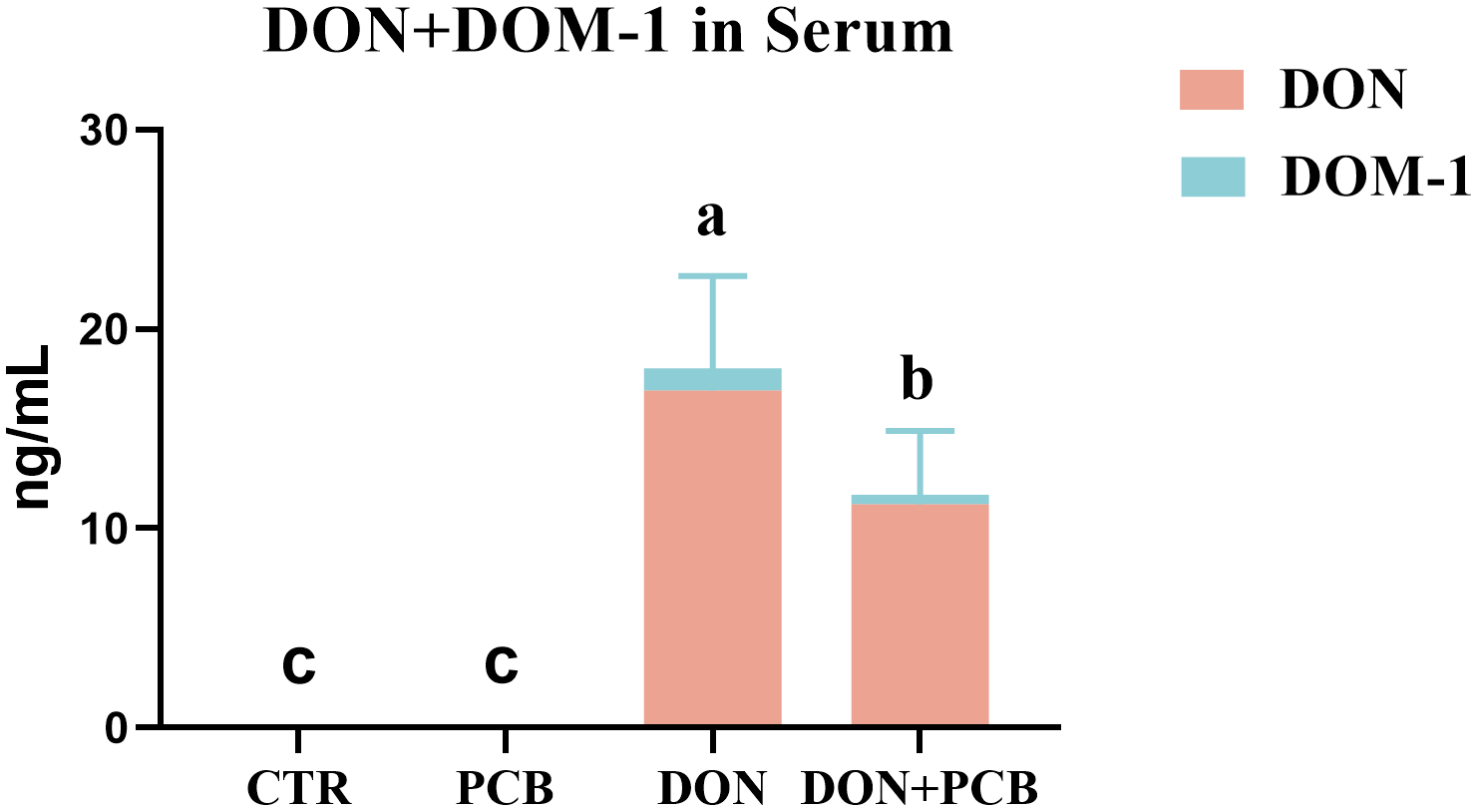
Effects of plant carbon black (PCB) on serum levels of deoxynivalenol (DON)+deepoxy-deoxynivalenol (DOM-1) in piglets challenged with DON. *N* = 6. CTR = basal diet; PCB = basal diet + 100 mg/kg PCB; DON = basal diet + 2.3 mg/kg DON; DON+PCB = basal diet + 2.3 mg/kg DON + 100 mg/kg PCB. ^a,b,c^Means within a row without a common superscripted letter are significantly different (*p* < 0.05).

DON exposure markedly elevated the serum content of DON+DOM-1 compared to the CTR and PCB group (**Figure 2**, *p* < 0.05). However, the supplement of PCB in DON-contaminated feed significantly decreased the serum content of DON+DOM-1 (*p* < 0.05), although it remained higher than that in the CTR and PCB groups (*p* < 0.05).

### 3.3 Morphometric parameters and goblet cells of the jejunum and ileum

**Figure 3:**
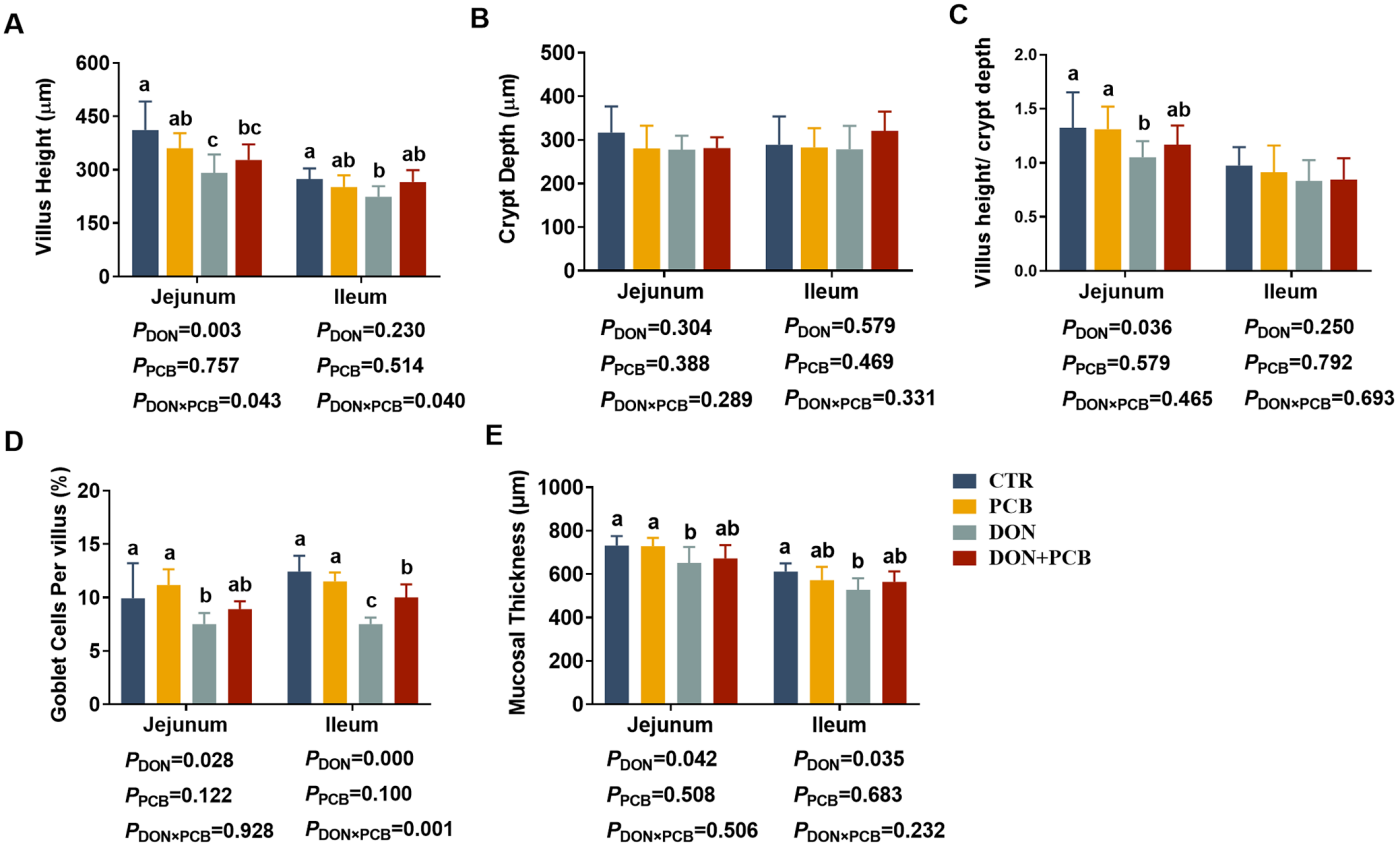
Effects of plant carbon black (PCB) on morphology, goblet cell, and mucosal thickness in the jejunum and ileum of piglets challenged with deoxynivalenol. **(A)** Villus height; **(B)** crypt depth; **(C)** villus height/crypt depth; **(D)** goblet cells per villus; **(E)** mucosal thickness. *N* = 6. CTR = basal diet; PCB = basal diet + 100 mg/kg PCB; DON = basal diet + 2.3 mg/kg DON; DON+PCB = basal diet + 2.3 mg/kg DON + 100 mg/kg PCB. ^a,b,c^Means within a row without a common superscripted letter are significantly different (*p* < 0.05).

As shown in **Figures 3A–E**, DON caused jejunal and ileal mucosal damage, with significant decreases in villus height, V/C, goblet cells, and mucosal thickness in the jejunum, and decreased villus height, goblet cells, and mucosal thickness in the ileum (*p* < 0.05). There was a significant DON×PCB interaction on jejunal villus height, ileal villus height, and ileal goblet cells (*p* < 0.05). The supplement of PCB prevented DON-mediated changes in V/C, jejunal mucosal thickness, and ileal mucosal thickness. Moreover, the DON+PCB group markedly increased goblet cell percentage in the ileum compared to the DON group (*p* < 0.05). No significant differences were noted in intestinal morphology between the PCB and CTR groups (*p* > 0.05).

### 3.4 Ultrastructure analysis of jejunum

**Figure 4:**
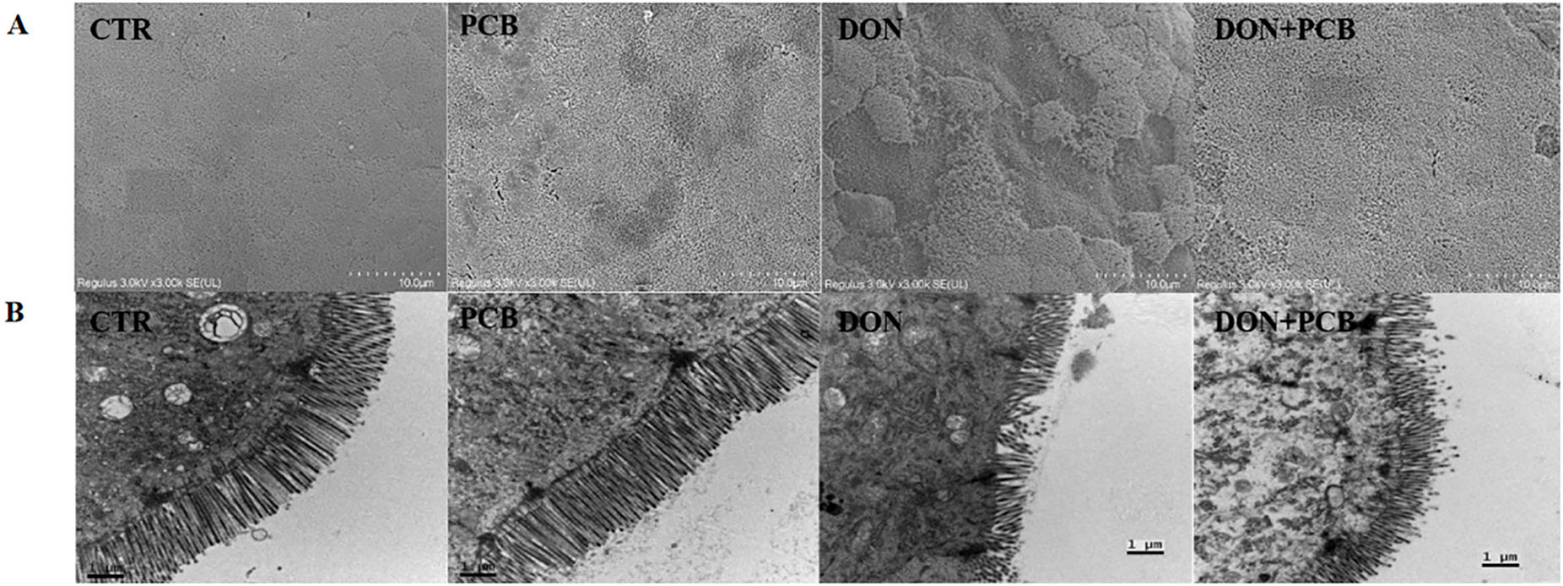
Effects of plant carbon black (PCB) on ultrastructure morphologies of jejunum in piglets challenged with deoxynivalenol. **(A)** Representative image of SEM observation. **(B)** Representative image of TEM observation. CTR = basal diet; PCB = basal diet + 100 mg/kg PCB; DON = basal diet + 2.3 mg/kg DON; DON+PCB = basal diet + 2.3 mg/kg DON + 100 mg/kg PCB.

SEM observations of the jejunum are shown in **Figure 4A**. CTR and PCB groups showed flat surfaces, closely arranged microvilli, and no lodging fractures. Exposure to DON led to the extensive loss of microvilli with a concave shape. Microvilli surrounding the affected area were sparsely distributed, with reduced tightness and lodging. Although the shedding of microvilli was also observed in the DON+PCB group, the situation was favorable compared with that of the DON group.

TEM (**Figure 4B**) revealed regular jejunal morphology with well-arranged microvilli, intact mitochondria membranes, and cristae in the CTR and PCB groups. In contrast, the DON group exhibited shortened and disordered microvilli arrangements, as well as fractured and swollen mitochondria with unclear and fragmented cristae. The supplementation of PCB alleviated the jejunal ultrastructural damage caused by DON.

### 3.5 Inflammation cytokines in the jejunum and ileum

**Figure 5:**
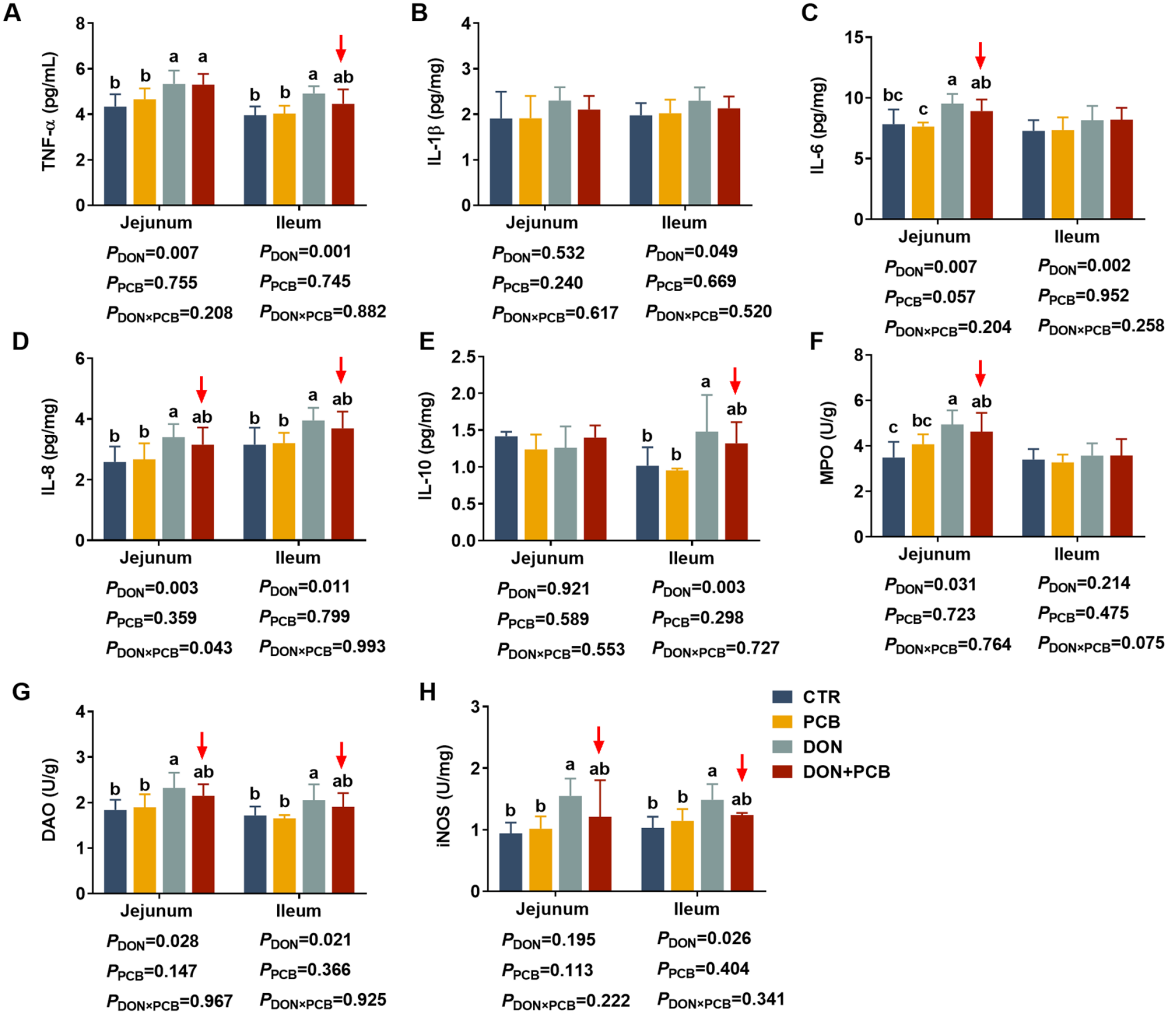
Effects of plant carbon black (PCB) on inflammation cytokines in the jejunum and ileum of piglets challenged with deoxynivalenol. **(A)** Tumor necrosis factor-α (TNF-α); **(B)** interleukin-1β (IL-1β); **(C)** interleukin-6 (IL-6); **(D)** interleukin-8 (IL-8); **(E)** interleukin-10 (IL-10); **(F)** myeloperoxidase (MPO); **(G)** diamine oxidase (DAO); **(H)** inducible nitric oxide synthase (iNOS). *N* = 6. CTR = basal diet; PCB = basal diet + 100 mg/kg PCB; DON = basal diet + 2.3 mg/kg DON; DON+PCB = basal diet + 2.3 mg/kg DON + 100 mg/kg PCB. ^a,b,c^Means within a row without a common superscripted letter are significantly different (*p* < 0.05).

As shown in **Figures 5A–H**, DON-challenged piglets showed increased TNF-α, IL-6, IL-8, MPO, and DAO in the jejunum, and increased TNF-α, IL-1β, IL-6, IL-8, IL-10, DAO, and iNOS in the ileum (*p* < 0.05). DON and PCB significantly interacted with IL-8 in the jejunum (*p* = 0.043) and tended to interact with MPO in the ileum (*p* = 0.075). PCB supplementation in a contaminated diet reversed the DON-induced increases of IL-6, IL-8, DAO, and iNOS in the jejunum and TNF-α, IL-6, IL-8, DAO, and iNOS in the ileum. Dietary supplementation with PCB alone showed no differences in intestinal inflammation indices compared to the CTR group (*p* > 0.05).

### 3.6 Antioxidant indices in jejunum and ileum

**Figure 6:**
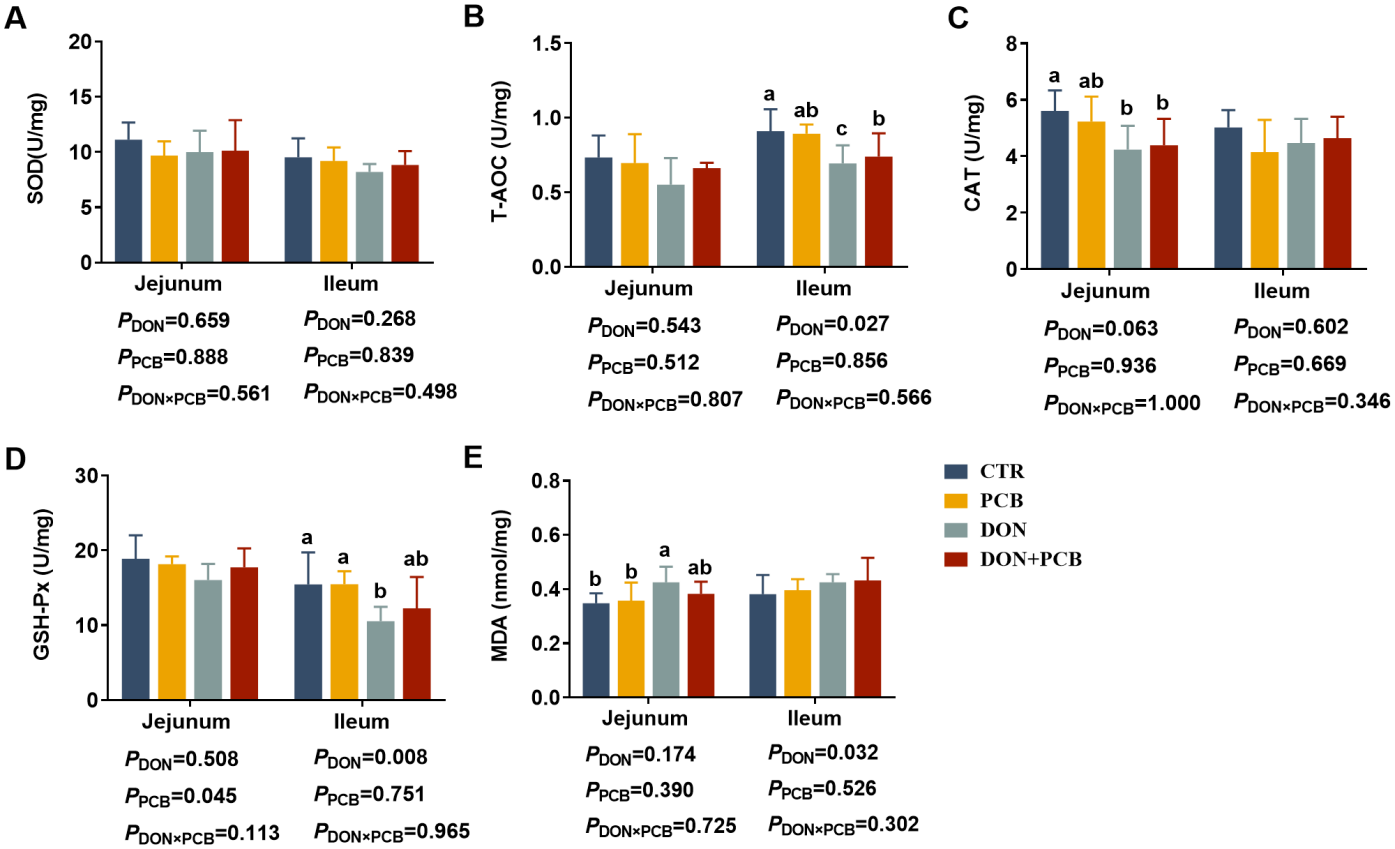
Effects of plant carbon black (PCB) on antioxidant indices in the jejunum and ileum of piglets challenged with deoxynivalenol. **(A)** Superoxide dismutase (SOD); **(B)** total antioxidant capacity (T-AOC); **(C)** catalase (CAT); **(D)** glutathione peroxidase (GSH-Px); **(E)** malondialdehyde (MDA). *N* = 6. CTR = basal diet; PCB = basal diet + 100 mg/kg PCB; DON = basal diet + 2.3 mg/kg DON; DON+PCB = basal diet + 2.3 mg/kg DON + 100 mg/kg PCB. ^a,b,c^Means within a row without a common superscripted letter are significantly different (*p* < 0.05).

As shown in **Figures 6A–E**, DON exposure decreased CAT, increased MDA in the jejunum, and decreased T-AOC and GSH-Px in the ileum (*p* < 0.05). Multiple comparisons showed that PCB supplementation to piglets challenged with DON prevented the reduction in GSH-PX and the increase in MDA in the ileum. PCB supplementation in the basal diet showed no differences in the indices of intestinal antioxidant capacity compared with the CTR group (*p* > 0.05).

### 3.7 mRNA expression levels of tight junction proteins, inflammatory cytokines, and ferroptosis-related genes in the jejunum and ileum

**Figure 7:**
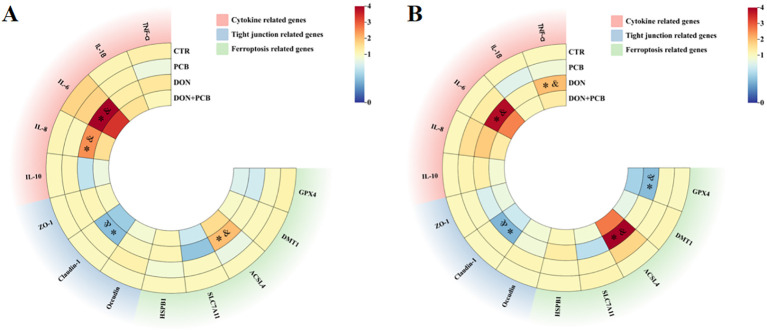
Effects of plant carbon black (PCB) on relative mRNA expression levels of tight junction, inflammatory cytokines, and ferroptosis-related genes in the jejunum **(A)** and ileum **(B)** of piglets challenged with deoxynivalenol. *N* = 6. CTR = basal diet; PCB = basal diet + 100 mg/kg PCB; DON = basal diet + 2.3 mg/kg DON; DON+PCB = basal diet + 2.3 mg/kg DON + 100 mg/kg PCB; TNF-α, tumor necrosis factor-α; IL-1β, interleukin-1β; IL-6, interleukin-6; IL-8, interleukin-8; IL-10, interleukin-10; ZO-1, zonula occludens-1; HSPB1, heat shock protein beta 1; SLC7A11, solute carrier family 7 member 11; ACSL4, long-chain acyl-CoA synthetase 4; DMT1, divalent metal transporter 1; GPX4, glutathione peroxidase 4. * means *p* < 0.05 as compared to the CTR group, & means *p* < 0.05 as compared to the PCB group.

The relative mRNA expression levels of tight junction proteins, inflammatory cytokines, and ferroptosis-related genes are shown in **Figure 7** (detailed information is shown in **Supplementary Table S4**). Compared to the CTR and PCB groups, DON treatment markedly increased the mRNA expression of IL-6, IL-8, and long-chain acyl-CoA synthetase 4 (ACSL4) and inhibited the mRNA expression of Claudin-1 in the jejunum (**Figure 7A**, *p* < 0.05). Meanwhile, DON exposure increased the mRNA expression of TNF-α, IL-6, and ACSL4 compared with the CTR and PCB groups. It decreased the mRNA expression of glutathione peroxidase 4 (GPX4) and Claudin-1 in the ileum (**Figure 7B**, *p* < 0.05). In contrast, no significant changes in the mRNA expression of cytokines, tight junctions, or ferroptosis-related genes were observed in the jejunum and ileum of the DON+PCB group compared to those of the CTR and PCB groups (*p* > 0.05).

### 3.8 SCFA concentration in cecal content

**Figure 8:**
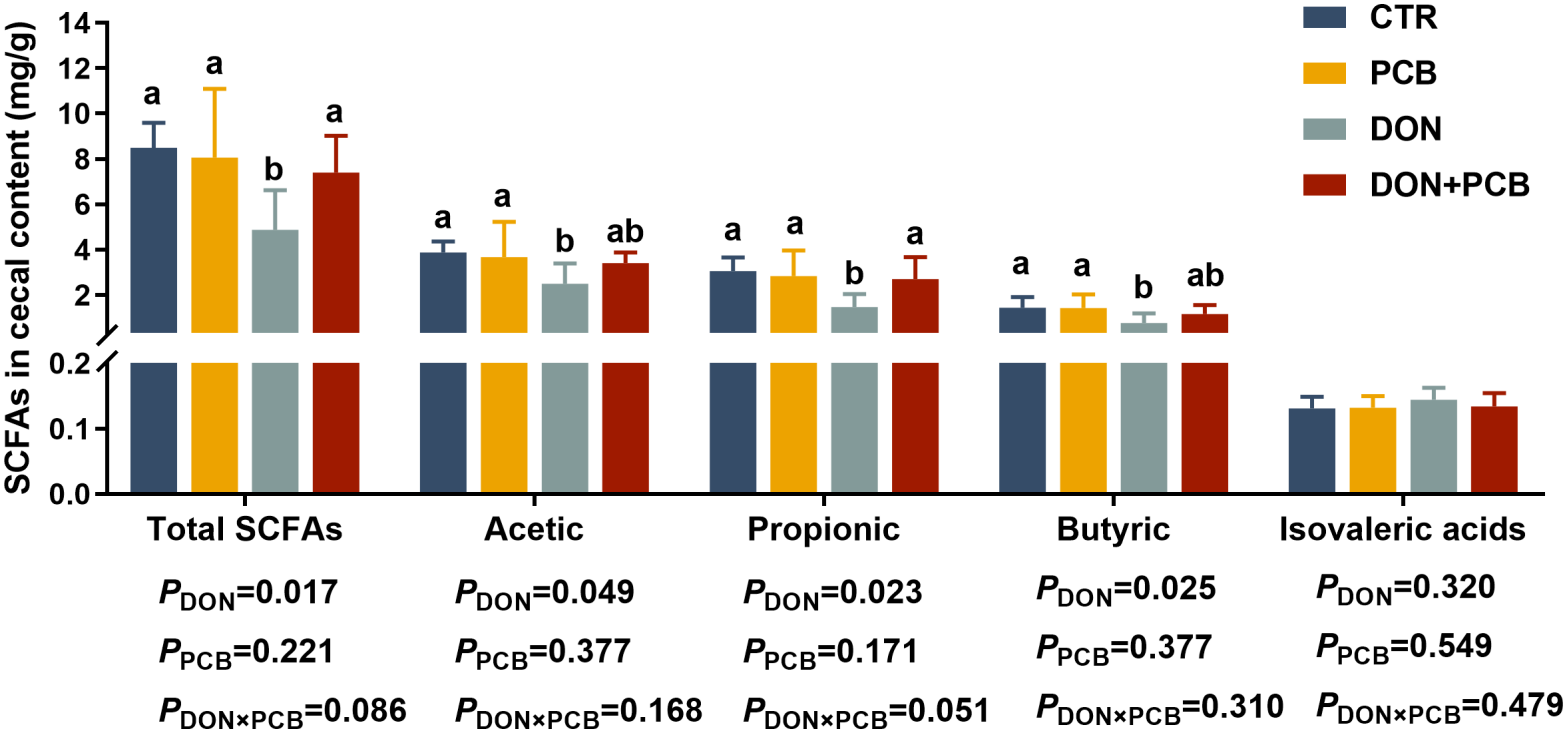
Effects of plant carbon black (PCB) on short-chain fatty acid in cecal content of piglets challenged with deoxynivalenol. *N* = 6. CTR = basal diet; PCB = basal diet +100 mg/kg PCB; DON = basal diet + 2.3 mg/kg DON; DON+PCB = basal diet + 2.3 mg/kg DON + 100 mg/kg PCB; SCFA, short chain fatty acids. ^a,b,c^Means within a row without a common superscripted letter are significantly different (*p* < 0.05).

The SCFA results are shown in **Figure 8**. Compared to non-challenged piglets, DON-challenged piglets showed a significant decrease in the cecal contents of total SCFAs, acetic, propionic, and butyric acids. There is an interactive trend between DON and PCB regarding total SCFAs (*p* = 0.086) and propionic acid content (*p* = 0.051). Multiple comparisons showed that PCB supplementation prevented the DON-induced decrease in total SCFAs, acetic, propionic, and butyric acids. Moreover, the DON+PCB group showed a marked increase in total SCFAs and propionic acid levels compared to the CTR group. PCB alone did not alter the SCFA concentrations in the cell content compared with the CTR group.

### 3.9 Cecal microbiota analysis

**Figure 9:**
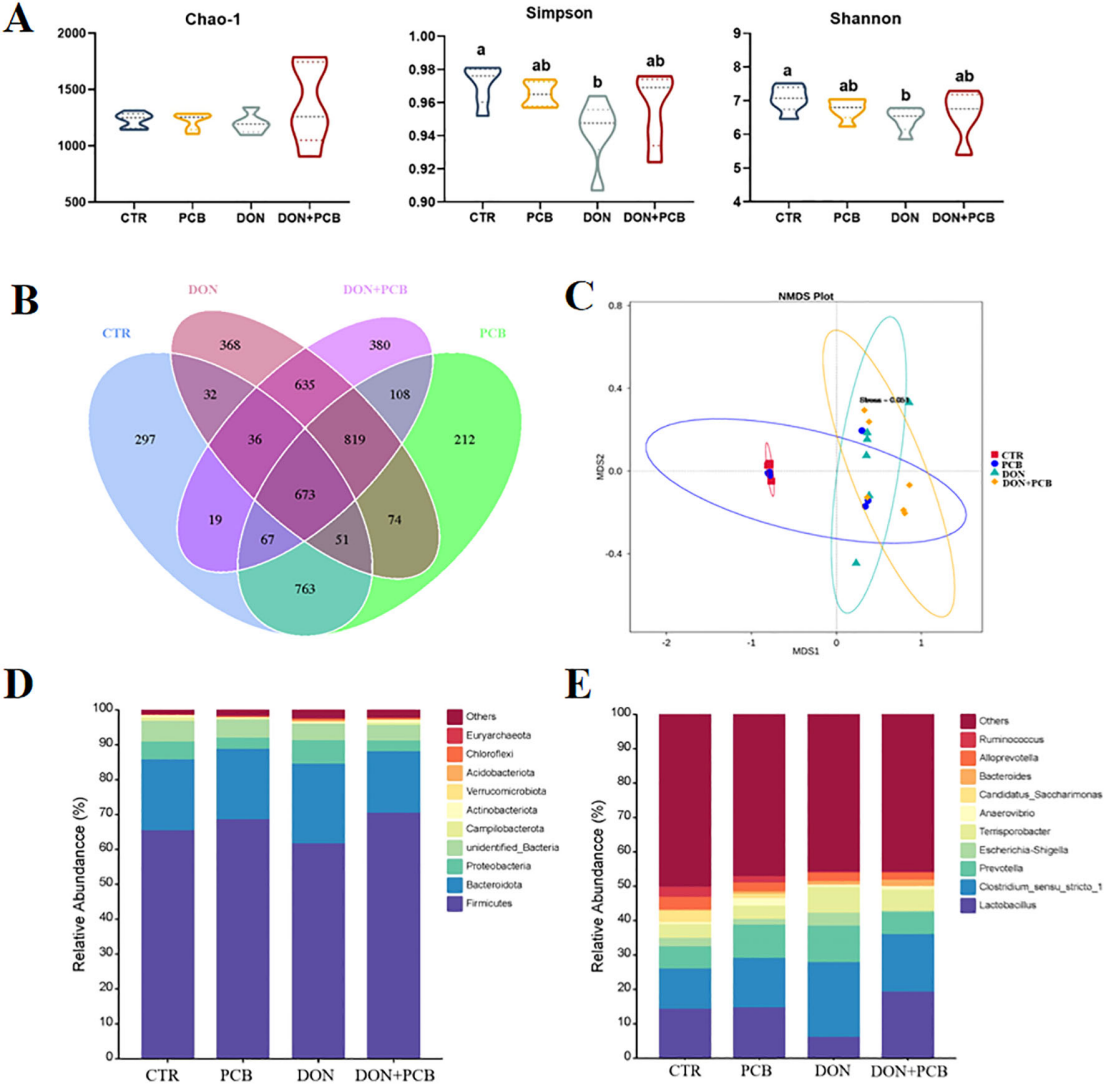
Effects of plant carbon black (PCB) on α- and β-diversity and composition of cecal microbiota in piglets challenged with deoxynivalenol. **(A)** α-diversity according to Chao-1, Simpson, and Shannon indices. **(B)** Venn diagram illustrating the overlap of the operational taxonomic units (OTUs). **(C)** β-diversity based on nonmetric multidimensional scaling (NMDS). **(D)** Relative abundance of cecal microbiota at the phylum level (top 10). **(E)** Relative abundance of cecal microbiota at the genus level (top 10). *N* = 6 per treatment. CTR = basal diet; PCB = basal diet +100 mg/kg PCB; DON = basal diet + 2.3 mg/kg DON; DON+PCB = basal diet + 2.3 mg/kg DON + 100 mg/kg PCB. ^a,b,c^Means within a row without a common superscripted letter are significantly different (*p* < 0.05).

As shown in **Figure 9A**, DON exposure markedly decreased the diversity of the microbiota (Simpson and Shannon indices). Still, it showed limited effects on microbiota richness (Chao-1 index) compared to the CTR group (*p* > 0.05). There were no significant differences in the α-diversity index after PCB supplementation in DON-challenged piglets compared to the CTR group (*p* > 0.05). As shown in **Figure 9B**, the piglets in the four treatments shared 673 OTUs and 297, 212, 368, and 380 unique OTUs in the CTR, PCB, DON, and DON+PCB groups, respectively. The nonmetric multidimensional scaling (NMDS) analysis showed that microbial profiles were separated between DON-challenged piglets and those not challenged (**Figure 9C**). The stress of the NMDS was 0.051, which ensured its reliability. *Bacteroidetes* and *Firmicutes* are the two most abundant bacterial phyla at the phylum level, comprising at least 84.64% of the total bacteria (**Figure 9D**). At the genus level, *Lactobacillus, Clostridium_sensu_stricto_1*, and *Prevotella* were the dominant genera in all four groups (**Figure 9E**).

**Figure 10:**
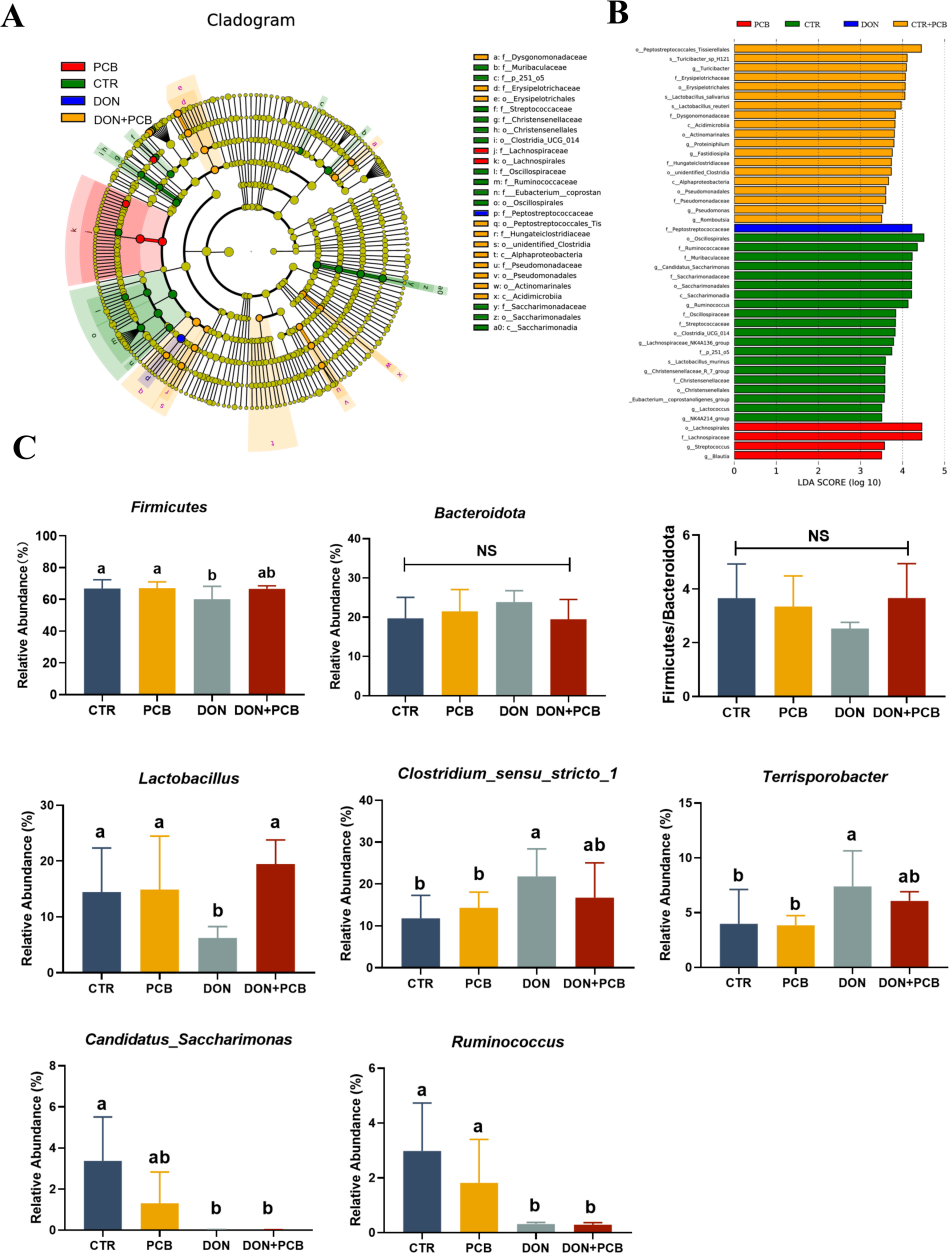
Effect of plant carbon black (PCB) on changed microbes of piglets challenged with deoxynivalenol. **(A)** LEfSe analysis with LDA scores (>3.5). **(B)** Significant differences of *Firmicutes*, *Bacteroidetes*, and their ratio. **(C)** Significant differences of cecal microbiota at the genus level (top 10). *N* = 6. CTR = basal diet; PCB = basal diet +100 mg/kg PCB; DON = basal diet + 2.3 mg/kg DON; DON+PCB = basal diet + 2.3 mg/kg DON + 100 mg/kg PCB. ^a,b,c^Means within a row without a common superscripted letter are significantly different (*p* < 0.05).

LEfSe analysis revealed the presence of 44 different bacterial taxa among the four groups (**Figure 10A**). As shown in **Figure 10B**, a significant decrease in the relative abundance of *Firmicutes* was observed in the DON group compared to that in the CTR group (*p* < 0.05), but this difference was not observed in the DON+PCB group (*p* > 0.05). The DON group showed decreases in the relative abundances of *Lactobacillus*, *Candidatus_Saccharimonas*, and *Ruminococcus* and increases in those of *Clostridium_sensu_stricto_1* and *Terrisporobacter* compared to the CTR group (*p* < 0.05, **Figure 10C**). The DON+PCB group showed decreases in the relative abundances of *Candidatus_Saccharimonas* and *Ruminococcus* (*p* < 0.05). Nonetheless, no significant differences were detected in the relative abundances of *Lactobacillus*, *Clostridium_sensu_stricto_1*, and *Terrisporobacter* compared to the CTR group (*p* > 0.05). In addition, a significant increase in the relative abundance of *Lactobacillus* was observed in the DON+PCB group compared to that in the DON group (*p* > 0.05).

### 3.10 Correlation analysis of gut microbiota and variables related to jejunum morphology, inflammation, antioxidant status, and cecal short chain-fatty acid

**Figure 11:**
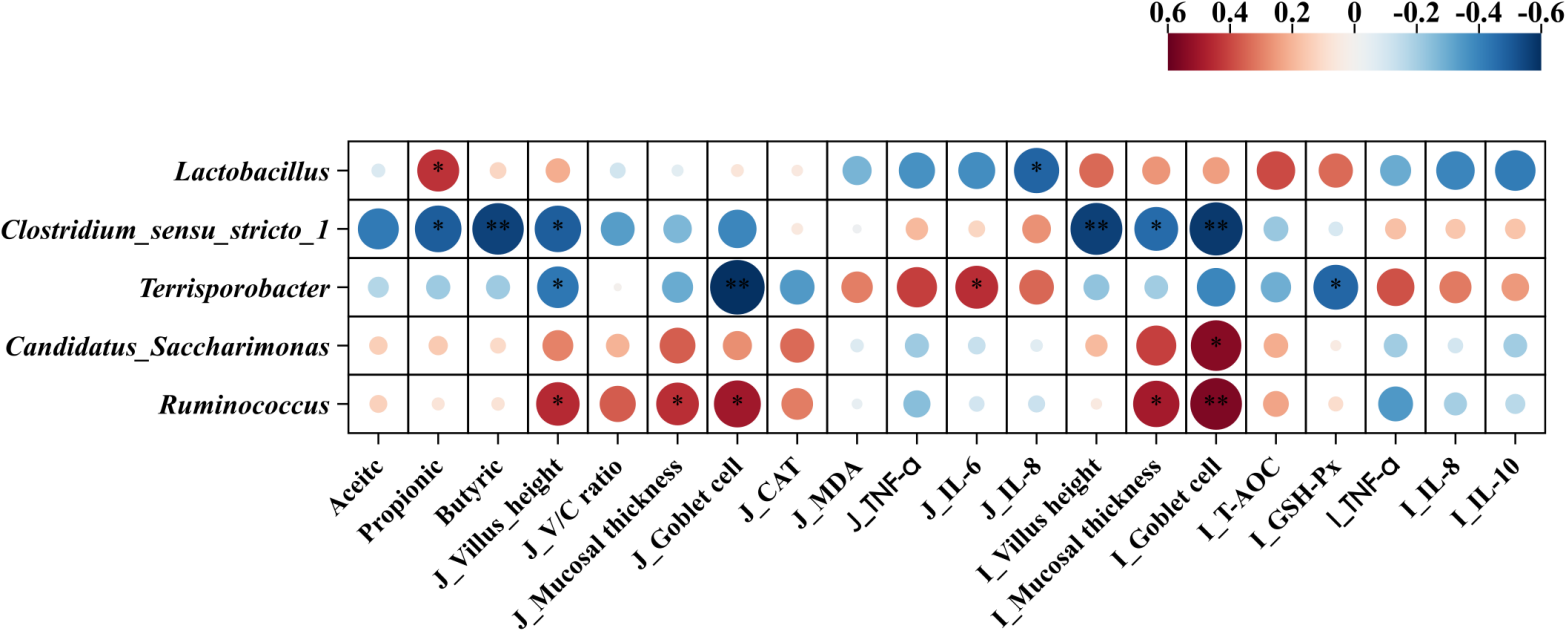
Pearson’s correlation coefficients between microbial genera and variables related to jejunum morphology, inflammation, antioxidant status, and short chain-fatty acid. *p < 0.05 and **p < 0.01. Red represents a significant positive correlation, and blue represents a significant negative correlation. J, jejunum; I, ileum; V/C ratio, villus height/crypt depth ratio; TNF-α, tumor necrosis factor-α; IL-6, interleukin-6; IL-8, interleukin-8; CAT, catalase; MDA, malondialdehyde; GSH-Px, glutathione peroxidase.

Pearson’s correlation coefficients between the main microbial genera and variables related to jejunal morphology, inflammation, antioxidant status, and cecal SCFAs are shown in **Figure 11**. fimmu.2024.1454530The relative abundance of *Lactobacillus* showed a positive correlation with propionic acid in the cecal content but was negatively associated with IL-8 in the jejunum. The relative abundance of *Clostridium_sensu_stricto_1* was negatively correlated with the villus height of the jejunum, villus height, mucosal thickness, goblet cells of the ileum, and propionic and butyric acid levels of the cecal content. The relative abundance of *Terrisporobacter* was positively correlated with IL-6 in the jejunum but showed negative correlations with villus height, goblet cells of the jejunum, and GSH-Px in the ileum. The relative abundance of *Candidatus_Saccharimonas* was positively correlated with goblet cells in the ileum. The relative abundance of *Ruminococcus* was positively correlated with the villus height, mucosal thickness, and goblet cells of the jejunum, as well as the mucosal thickness and goblet cells of the ileum.

## 4 Discussion

DON is a potent mycotoxin prevalent in the grains used in swine feed. Exposure in pigs poses substantial risks, including reduced feed intake, vomiting, diarrhea, and disruption to intestinal morphology and functions (8, 27). Mitigation strategies against DON remain less developed because of its high polarity, lack of a coplanar structure, and low molecular weight (15). PCB, a new feed additive approved by the Ministry of Agriculture of China in 2020, primarily comprises porous carbon. With an adsorption total pore volume of 1.28 cm^3^/g, a specific surface area of 1,313.40 m^2^/g, and a total adsorption average pore width of 3.56 nm, PCB has an excellent adsorption potential for DON (21).

Before assessing the *in vivo* intestinal effects, we evaluated the adsorption effects of PCB on DON *in vitro*. These results confirmed DON’s effective and durable binding to PCB across a range of initial concentrations under simulated gastrointestinal conditions. Although the maximum adsorption capacity was not measured in the present study, available data showed that PCB has previously performed better than ordinary activated carbon, with an adsorption rate of 91.92% vs. 59% at a DON concentration of 10 μg/mL (16). Our results are similar to those reported by Ying et al., who prepared a novel porous carbon adsorbent from soybean dregs and achieved a DON removal efficiency of 88.31% (17). However, the present study’s adsorption efficiency declined under simulated gastrointestinal conditions, indicating competitive and non-specific interactions between enzymes, bile salts, and mineral loads *in vivo*. Therefore, further *in vivo* experiments are required to verify the DON adsorption efficacy of PCB. Serum DON and its metabolite DOM-1 are two specific parameters used to evaluate the efficiency of DON adsorption by mycotoxin binders (28). In the present study, a 0.1% PCB inclusion conferred a reduction in DON bioavailability in DON-challenged piglets, as shown by the 35.14% reduction in serum levels of DON+DOM-1. Although the reduction of DON in the serum was not as high as *in vitro*, partial sequestration occurred in a dynamic gastrointestinal environment, limiting systemic toxin distribution. Similar results were reported by Devreese et al., who found that activated carbon significantly reduced the absorption and oral availability of DON, as evidenced by decreased serum DON levels in pigs (29) and broiler chickens (30).

Intestinal morphology is an important indicator of nutrient absorption ability and intestinal barrier function in piglets (31). In the present study, DON exposure shortened villi, reduced the V/C ratio, disrupted and shredded microvilli, and decreased mucosal thickness. Our findings are consistent with previous studies on porcine intestinal epithelial cells and experimental pig models (32, 33). Liao et al. noted that DON markedly decreases the crypt depth in pigs’ jejunum and ileum (34). These morphological changes are associated with reduced nutrient digestion and absorption, slowing piglet growth (34, 35). These results are consistent with our previous study’s decrease in body weight and average daily gain (36).

Additionally, we found that exposure to DON increased the mucosal levels of DAO and decreased the mRNA expression of Claudin-1, which suggests an augmented paracellular permeability and impairment of the intestinal epithelial barrier (37). In the present study, supplementation with 0.1% PCB attenuated DON-induced damage to intestinal morphology and barrier function, indicating that PCB was effectively bound to DON and decreased contact with the intestines. The reduced serum levels of DON+DOM-1 confirmed this hypothesis. In addition to these binding effects, Wang et al. noted that PCB promoted intestinal mucosal development by increasing serum IGF-1 levels and mucosal SIgA content in weaning piglets (21).

In addition to disrupting the morphology and permeability, DON triggers aberrant inflammation by permeating the intestinal epithelium. Pro-inflammation cytokines, such as TNF-α, IL-6, and IL-8, are produced by intestinal epithelial cells, reflecting the presence and intensity of the inflammation reaction. MPO is a specific polymorphonuclear leukocyte enzyme abundant in neutrophils and used as a marker to evaluate intestinal inflammation. In our present study, DON exposure increased the protein and mRNA expression of inflammation cytokines (TNF-α, IL-6, and IL-8) and MPO contents in the jejunum or ileum of piglets; these increases can cause chemokine signaling, immune cell recruitment, and feed-forward damage without adequate countermeasures (38). These results follow previous studies that reported that DON exposure upregulated the mRNA or protein expression of pro-inflammation cytokines such as TNF-α and IL-6 in mice (37) and chickens (39). IL-10 is an anti-inflammatory cytokine that is known to promote the resolution of inflammation.

In the present study, DON increased IL-10 content in the ileum, suggesting that the mucosal immune system attempts to control inflammation by a compensatory increase in IL-10. Dietary PCB supplementation effectively mitigated DON-induced jejunal and ileal inflammatory responses by decreasing cytokine mRNA and protein expression to basal levels. Reducing these mediators helps mitigate neutrophil and macrophage infiltration and potential secondary T-cell reactions, thereby reducing further damage to membrane integrity and absorptive capacity (40).

Several studies have demonstrated that DON accelerates the production of free radicals, damages mitochondrial function, induces lipid peroxidation, and reduces the activity of antioxidant enzymes (34, 41, 42). Alterations in the antioxidant system lead to oxidative stress, which may be one of the main causes of intestinal inflammation and destruction of the epithelial mucosal structure (42). Consistently, the results of the present study showed that DON-challenged piglets had reduced T-AOC, GSH-Px, and CAT levels but increased MDA levels in the intestinal mucosa. T-AOC, GSH-Px, and CAT are important indices of the antioxidant defense system, and a decrease in their levels indicates a decrease in the free radical scavenging ability and antioxidant levels. MDA is a product of peroxidized polyunsaturated fatty acids, and increased MDA levels reflect lipid peroxidation. A study by Zhang et al. showed contradictory results (43), with the exposure of piglets to DON upregulating T-AOC and GSH-Px levels. These differences may have resulted from differences in exposure time, DON levels, or host health status, which require further study. In the present study, PCB supplementation significantly suppressed the alterations in GSH-Px, T-AOC, and MDA levels induced by DON, indicating that PCB mitigated the DON-induced oxidative stress by reducing the depletion of peroxide-targeting enzymes and prevented the accumulation of lipid oxidation. A previous study consistently found that activated carbon adsorbents markedly increased GSH-Px and decreased MDA in the liver of rats treated with DON and were more effective than Egyptian montmorillonite (44).

Ferroptosis, a recently discovered form of programmed cell death, is distinguished by extensive lipid peroxidation triggered by iron overload and ROS production (45). In the present study, DON exposure decreased the mRNA expression of GPX4 in the ileum and increased the mRNA expression of ACSL4 in the jejunum and ileum. ACSL4 and GPX4 are the key factors and regulators of ferroptosis. ACSL4 promotes ferroptosis and regulates ferroptosis sensitivity, whereas GPX4 inhibits ferroptosis (46). The results of this study indicate that ferroptosis influences DON-induced intestinal injuries. Similar to our results, Liu et al. revealed that DON alters ferroptosis-related gene expression in IPEC-J2 cells and porcine duodenum (12). Notably, PCB supplementation prevented an increase in ACSL4 and decreased GPX4 mRNA expression in the jejunum and ileum, illustrating that PCB protected the intestine from DON-induced ferroptosis.

The intestinal microbiota maintains intestinal function and health (47). DON exposure can disrupt the microbiota balance in pigs (48, 49). In the present study, DON reduced the diversity of piglet microbiomes, indicating decreased stability of the microbiota system. Jia et al. consistently noted that DON decreased the jejunum microbiota diversity in broilers and pigs (50). *Bacteroidetes* and *Firmicutes* are the two dominant bacterial phyla in piglets. In this study, the relative abundance of *Firmicutes* was markedly decreased, and the *Firmicutes/Bacteroidetes* ratio tended to decrease in DON-treated piglets. The reduction is thought to be linked to poor oxidative stress response and high inflammation and infection risk (51, 52). The current study provides evidence for this hypothesis by observing elevated levels of inflammatory cytokines and reduced levels of antioxidant enzymes in the DON group. Similar to our results, Li et al. found that DON exposure decreased the relative abundance of *Firmicutes* and increased the abundance of *Bacteroidetes* in the ileum and colon of piglets (53). At the genus level, DON decreased the relative abundance of *Lactobacillus* and increased that of *Clostridium sensu stricto 1. Clostridium sensu stricto 1* is an opportunistic pathogen that crucially influences intestinal inflammation occurrence and reduces SCFA content (54, 55). *Lactobacillus* is a beneficial bacterium that positively affects gut mucosal immune barrier function and SCFA production (56). The decreased *Lactobacillus* and increased *Clostridium sensu stricto 1* explain the reduction of SCFA in the cecal contents of piglets challenged with DON. The PCB treatment partially reversed the alteration in the gut microbiota in DON-challenged piglets. In addition to reducing the negative effects on bacterial flora by adsorbing DON, studies have shown that PCB can directly act on bacterial flora to improve their structure. Wang et al. noted that PCB improved the bacterial community by decreasing the number of *E. coli* in the cecum at a dose of 500 mg/kg in piglets (21). In fattening pigs fed ordinary diets, 0.3% charcoal derived from bamboo showed increased lactic acid bacteria and decreased *coliform bacteria* and *Salmonella* spp (57).

## 5 Conclusion

In conclusion, the results of the present study demonstrate the protective effects of 0.1% PCB against various intestinal toxic manifestations in DON-challenged piglets. Beyond demonstrating high adsorption efficacy *in vitro*, the supplementation with PCB also helped protect intestinal morphology, reduce inflammation and oxidative stress, and improve the intestinal microbiota in DON-challenged piglets. No negative effects on these intestinal parameters were observed in piglets supplemented with PCB alone.

## Data Availability

The original contributions presented in the study are included in the article/**Supplementary Material**. Further inquiries can be directed to the corresponding authors.
