# Supplementary material for: Mitigation effects of plant carbon black on intestinal morphology, inflammation, antioxidant status, and microbiota in piglets challenged with deoxynivalenol
Source: Front Immunol. 2024 Sep 9;15:1454530. doi: 10.3389/fimmu.2024.1454530 (PMC11416923; doi:10.3389/fimmu.2024.1454530)
Supplement: Supplementary file 1 [file Table1.docx]

**Supplementary materials**

**Supplementary method S1 Preparation of artificial gastric juice and artificial intestinal juice**

Preparation of artificial gastric juice: Dissolve 2 g NaCl and 3.2 g pepsin in 500 mL water, then add 5 mL HCl, make up to 1000 mL with water, and adjust the pH to 2.0 with 0.1 M NaOH.

Preparation of artificial intestinal juice: Dissolve 6.8 g KH2PO4 in 500 mL water and adjust the pH to 6.8 with 0.1 M NaOH. Dissolve 10 g trypsin in water, mix with the KH2PO4 solution, dilute to 1000 mL with water, and adjust the pH to 6.5 with 0.1 M NaOH and 0.1 M HCl.

**Supplementary Table S1** Ingredients and nutrients composition of the basal diet (as fed basis)

| Ingredients | Percentage, % | Nutrition component | Content |
| --- | --- | --- | --- |
| Corn | 54.75 | Crud protein, % | 18.46 |
| Soybean meal | 18 | Digestible energy, MJ/Kg | 3400 |
| Extruded soybean | 8 | Lysine, % | 1.28 |
| Whey powder | 5 | Methionine, % | 0.50 |
| Fish meal | 5 | Threonine, % | 0.89 |
| brown sugar | 5 | Calcium, % | 0.75 |
| Soybean oil | 1 | Total phosphorus, % | 0.64 |
| NaCl | 0.25 | Available phosphorus, % | 0.45 |
| Choline chloride 50% | 0.1 |  |  |
| Premix^b^ | 3 |  |  |

Premix provided per kg diet: Fe, 100 mg; Cu, 20 mg; Zn, 100 mg; Mn, 60 mg; I, 0.35 mg; Se, 0.4 mg; Vitamin A, 12000 IU; Vitamin D3, 300 IU; Vitamin E, 130 mg; Vitamin K3, 5 mg; Vitamin B1, 4 mg; Vitamin B2, 15 mg; Vitamin B6, 7 mg; Vitamin B12, 50 μg; Nicotinamide, 50 mg; pantothenic acid, 30 mg; biotin, 0.15 mg; and folic acide, 1.5 mg.

**Supplementary Table S2** Mycotoxin composition of experimental diets

| **Mycotoxin(μg/kg)** | CTR^a^ | PCB | DON | DON+PCB |
| --- | --- | --- | --- | --- |
| Aflatoxin B1 | 1.08 | 1.92 | 1.28 | 1.18 |
| Zearalenone | 21.8 | 42.7 | 27.2 | 37.8 |
| Deoxynivalenol | 137 | 167 | 2285 | 2333 |

^a^ CTR, basal diet; PCB, basal diet + 0.1% Plant carbon black(PCB); DON, 2.3 mg/kg DON-contaminated basal diet; DON+PCB, 2.3 mg/kg DON-contaminated basal diet(DON)+0.1% PCB.

**Supplementary Table S3 Primers for real-time PCR in this study**

| Gene symbol | Accession number | Forward | Reverse |
| --- | --- | --- | --- |
| GAPDH | NM_001206359 | ACTCACTCTTCCACTTTTGATGCT | TGTTGCTGTAGCCAAATTCA |
| IL-1β | NM_001005149 | GAGCTGAAGGCTCTCCACCTC | ATCGCTGTCATCTCCTTGCAC |
| IL-6 | NM_214399 | GGCAAAAGGGAAAGAATCCAG | CGTTCTGTGACTGCAGCTTATCC |
| IL-8 | NM_213867 | GCTCTCTGTGAGGCTGCAGTTC | AAGGTGTGGAATGCGTATTTATGC |
| IL-10 | NM_214041 | GGCCCAGTGAAGAGTTTCTTTC | CAACAAGTCGCCCATCTGGT |
| TNF-α | NM_214022 | ACTGCACTTCGAGGTTATCGG | GGCGACGGGCTTATCTGA |
| ZO-1 | XM_003480423.3 | ATCTCGGAAAAGTGCCAGGA | CCCCTCAGAAACCCATACCA |
| Claudin-1 | NM_001244539.1 | TATGACCCCATGACCCCAGT | GCAGCAAAGTAGGGCACCTC |
| Occudin | NM_001163647.2 | CATGGCTGCCTTCTGCTTCATTGC | ACCATCACACCCAGGATAGCACTCA |
| HSPB1 | NM_001007518 | CTCGGAGATCCAGCAGACT | TCGTGCTTGCCCGTGAT |
| SLC7A11 | XM_021101587.1 | GCCTTGTCCTATGCTGAGTTG | GTTCCAGAATGTAGCGTCCAA |
| ACSL4 | NM_004458.1 | GAGGGAGGCCATCGAGAATG | GACCAGGTGCTGGGATTTGT |
| DMT1 | NM_001128440.1 | GCAGGTGGTTGACGTCTGTA | CACGCCCCCTTTGTAGATGT |
| GPX4 | NM_214407.1 | CTGTTCCGCCTGCTGAA | ACCTCCGTCTTGCCTCAT |

**Supplementary Table S4** Effects of plant carbon black(PCB) on relative mRNA expression levels of tight junction, inflammatory cytokines and ferroptosis-related genes in jejunum and ileum of piglets challenged with deoxynivalenol.

| Items | Groups | | | | SEM | P value | | |
| --- | --- | --- | --- | --- | --- | --- | --- | --- |
|  | CTR | PCB | DON | DON+PCB |  | DON | PCB | DON×PCB |
| Jejunum |  |  |  |  |  |  |  |  |
| GPX4 | 1.22 | 1.03 | 0.75 | 0.80 | 0.13 | 0.189 | 0.785 | 0.627 |
| DMT1 | 1.15 | 1.04 | 1.00 | 0.97 | 0.16 | 0.762 | 0.852 | 0.909 |
| ACSL4 | 1.06b | 0.87b | 2.01a | 1.60ab | 0.17 | 0.013 | 0.345 | 0.725 |
| SLC7A11 | 1.06 | 1.00 | 0.51 | 0.73 | 0.11 | 0.071 | 0.712 | 0.511 |
| HSPB1 | 1.06 | 0.92 | 1.12 | 1.10 | 0.07 | 0.401 | 0.589 | 0.674 |
| IL-1β | 1.15 | 1.21 | 1.30 | 1.52 | 0.15 | 0.455 | 0.660 | 0.802 |
| IL-6 | 1.71b | 1.74b | 5.50a | 3.38ab | 0.67 | 0.044 | 0.410 | 0.400 |
| IL-8 | 1.14b | 1.07b | 2.48a | 1.56ab | 0.22 | 0.034 | 0.230 | 0.303 |
| IL-10 | 1.06 | 1.11 | 0.68 | 0.89 | 0.09 | 0.111 | 0.479 | 0.670 |
| TNF-α | 1.25 | 0.89 | 1.43 | 1.09 | 0.14 | 0.528 | 0.244 | 0.979 |
| ZO-1 | 1.04 | 1.10 | 1.11 | 0.99 | 0.08 | 0.902 | 0.856 | 0.619 |
| Claudin-1 | 1.02a | 1.07a | 0.48b | 0.55ab | 0.10 | 0.008 | 0.746 | 0.947 |
| Occudin | 1.02 | 1.23 | 1.03 | 0.93 | 0.08 | 0.376 | 0.740 | 0.329 |
| Ileum |  |  |  |  |  |  |  |  |
| GPX4 | 1.02a | 1.03a | 0.47b | 0.58ab | 0.10 | 0.014 | 0.736 | 0.784 |
| DMT1 | 1.15 | 1.05 | 1.01 | 0.85 | 0.13 | 0.538 | 0.647 | 0.915 |
| ACSL4 | 1.03a | 1.70a | 4.34b | 2.69ab | 0.45 | 0.012 | 0.526 | 0.146 |
| SLC7A11 | 1.16 | 1.14 | 0.66 | 0.92 | 0.14 | 0.232 | 0.963 | 0.713 |
| HSPB1 | 1.04 | 1.05 | 1.33 | 1.08 | 0.06 | 0.218 | 0.376 | 0.303 |
| IL-1β | 1.10 | 0.82 | 1.15 | 1.06 | 0.10 | 0.511 | 0.410 | 0.661 |
| IL-6 | 1.16b | 1.23b | 3.16a | 2.14ab | 0.45 | 0.025 | 0.576 | 0.369 |
| IL-8 | 1.06 | 1.56 | 1.84 | 1.34 | 0.19 | 0.470 | 0.994 | 0.208 |
| IL-10 | 1.05 | 1.16 | 1.26 | 0.93 | 0.15 | 0.976 | 0.732 | 0.498 |
| TNF-α | 1.06b | 0.91b | 2.01a | 1.34ab | 0.19 | 0.077 | 0.278 | 0.490 |
| ZO-1 | 1.04 | 1.01 | 0.78 | 0.88 | 0.06 | 0.115 | 0.754 | 0.599 |
| Claudin-1 | 1.01a | 1.07a | 0.44b | 0.73ab | 0.10 | 0.021 | 0.335 | 0.536 |
| Occudin | 1.01 | 1.13 | 0.91 | 0.92 | 0.06 | 0.187 | 0.591 | 0.628 |
